# Establishing a tumour bank: banking, informatics and ethics

**DOI:** 10.1038/sj.bjc.6601678

**Published:** 2004-03-02

**Authors:** S J Qualman, M France, W E Grizzle, V A LiVolsi, C A Moskaluk, N C Ramirez, M K Washington

**Affiliations:** 1 Columbus Children's Hospital, 700 Children's Drive, Columbus, OH 43205, USA; 2 Department of Pathology ZRB 422, University of Alabama at Birmingham, 1530 Third Avenue, South, Birmingham, AL 35294-0007, USA; 3 Surgical Pathology Section, Founders Room 6.039, University of Pennsylvania Medical Center, 3400 Spruce Street, Philadelphia, PA 19194, USA; 4 Department of Pathology and Department of Biochemistry and Molecular Genetics, The University of Virginia Health System, PO Box 800214, Charlottesville, VA 22908, USA; 5 Department of Pathology, The Ohio State University, E412 Doan Hall, 410 West 10th Ave., Columbus, OH 43210-1228, USA; 6 Department of Pathology, Vanderbilt University Medical Center, 1161 21st Avenue, South, Nashville, TN 37232-2561, USA

**Keywords:** tissue banking, informatics, quality control, patient confidentiality

## Abstract

The six divisions of the Cooperative Human Tissue Network in the USA bank and distribute tens of thousands of tissue specimens to researchers annually. Major operational concerns include: maintaining tissue integrity, managing informatics, and protecting patient confidentiality. Increasing molecular genetics testing is also resulting in an increased demand for high-quality nucleic acids.

This review will deal with the broad concepts of tumour banking and informatics as practiced at the Biopathology Centers (BPC) located at the Children's Research Institute, Columbus, Ohio, USA. The BPC provides banking and informatics services to a variety of cancer cooperative groups under the tutelage of the Cooperative Human Tissue Network, including: Children's Oncology Group (COG), Gynecologic Oncology Group (GOG), and Childhood Cancer Survivor Study (CCSS). The BPC deals with patient specimens that span the age range of newborns to the elderly; procuring tumour types that include the spectrum of paediatric solid tumours, selected adult gynaecologic tumours (ovarian, cervical, uterine), and any second malignant (solid) neoplasm in childhood cancer survivors. In the last decade, the BPC has served nearly 85 000 tumour or related tissue specimens to over 300 different investigators. These specimens are linked to patient clinical outcome, because of the BPC's alliance with the aforementioned cooperative groups.

The National Cancer Institute Cooperative Human Tissue Network (CHTN) itself was founded 15 years ago ‘to stimulate, for the good of the public, cooperative efforts to collect and distribute human tissues and to thereby facilitate research utilising those tissues’. These activities are expected to encourage basic, developmental, and translational studies in many areas of cancer research including molecular biology, immunology, and genetics. The six institutions comprising the CHTN currently include the University of Pennsylvania (V LiVolsi, PI), University of Alabama at Birmingham (W Grizzle, PI), The Ohio State University (N Ramirez, PI), Columbus Children's Hospital (S Qualman, PI), The University of Virginia (C Moskaluk, PI) and Vanderbilt University (M Washington, PI). The CHTN does not provide fresh or frozen tissues with known pathogens or foetal tissues for research purposes. Collectively, these institutions supply an estimated 80 000 research specimens to hundreds of investigators annually. The CHTN as a whole is devoted primarily to the prospective procurement of tissue for immediate disbursal to investigators, rather than banking of specimens, but many of the logistical, informatic, and ethical issues are the same.

## BANKING

Many of the details of banking and informatics discussed below can be found in a recently published book chapter (Qualman *et al*, 2003), which ties these needs to the advancement of molecular research.

The design of tumour banks should be such that significant effort is devoted to obtaining data on clinical outcomes (Grizzle and Sexton, 1999), which permits investigators to know that such data are available for analysis as they pursue their molecular studies on bank-derived specimens. Tumour banking and its associated inventory informatics have been recognised for over a decade (Naber *et al*, 1992; Naber, 1996) as needed tools to advance the science of molecular testing; however, it has only been in the last 2–3 years that the linkage to clinical outcomes has been seen as crucial to achieving this goal (Naber *et al*, 1992). It is estimated that by the year 2005 (Farkas *et al*, 1996), as much as 10% of clinical laboratory tests will be based on RNA or DNA analysis. These efforts (Abati and Liotta, 1996) will only be successful with the logical application of tumour banking and its associated informatics systems as the translational bridge linking new molecular information to its clinical significance.

### Specimen transport

Cooperative group affiliated institutions must send specimens to the BPC through air-express couriers within 24 h of procurement, using dual-chamber kits, which allow for simultaneous delivery of specimens at ambient and deep-frozen temperatures (Qualman *et al*, 2003). In those situations, the BPC provides institutions with a courier account number to encourage rapid shipment. Shipments made via overnight carrier must be packaged to conform with International Air Transport Association (IATA) Section 1.5.0.2 (http://www.iata.org/index.htm). Researchers receiving specimens are billed for shipping charges and an additional specimen-processing fee associated with the types and numbers of specimens served. A toll-free telephone number is maintained for contacts with both institutions submitting specimens and investigators.

### Storage facility

Most tissues at the BPC are stored in liquid nitrogen vapour-phase freezers, because specimens kept at approximately −170°C are of higher integrity than those maintained at higher temperatures and show less desiccation (Grizzle *et al*, 1998; Grizzle and Sexton, 1999). Freezers (−80°C) are used for short-term tissue storage (1 week or less) and storage of blood and serum whereas −20°C freezers are used for reagent storage. Manual defrost units are preferred as the freeze–thaw cycles of automatic defrost units can degrade the quality of biologics and reagents.

Paraffin blocks, scrolls, and slides are best preserved in vacuum-sealed mylar bags with a commercial oxygen absorber at 4°C. Paraffin slides may also be dipped in molten paraffin prior to storage to preserve tissue antigenicity (Camp *et al*, 2000). The BPC utilises a cold room (walk-in cooler) (4°C) for storing its vacuum-sealed unstained slides and paraffin blocks.

Touch preparations, taken and fixed from fresh tumour specimens, were once standard submissions to the BPC, but are now best performed from segments of snap-frozen tissue stored at the bank. These can then be fixed and sent to the investigator with relatively good preservation of antigenicity and morphology.

Long-term storage (more than 1 week) of specimens in vapour-phase liquid nitrogen freezers is done in small segments (e.g. 5 g or less) in plastic histology cassettes or mega-cassettes with the specimens wrapped in aluminium foil to minimise desiccation. OCT-embedded specimens in cryomoulds should likewise be wrapped in foil. Glass vials and pop-top plastic vials are not adequate storage containers for vapour-phase liquid nitrogen temperatures, as they may readily break or pop open (Grizzle and Sexton, 1999). Screw-cap cryovials work well for storage of serum or urine.

### Quality assessments

Central morphologic reviews are performed on the formalin-fixed tissues sent via the dual-chamber kits which represent the mirror-image of samples of snap-frozen tumour and normal adjacent tissue also included in the kit. The histologic analyses include such parameters as: assessment of tumour diagnosis, percent tumour, percent stroma, and percent necrosis.

In a BPC sample of 7000 paediatric and gynaecologic tumours examined over a 5-year period, a diagnostic discrepancy rate of approximately 10% between central and institutional diagnoses was identified. Although this discrepancy rate might at first appear to be quite high, these data in the main reflect sampling problems identified in tissue aliquots labelled by the submitting institution as ‘normal’ or ‘tumour’ which were not reflective of the aliquot's content (e.g. tissue totally necrotic, normal tissue contaminated by tumour, etc.). Less than 2% of cases contained true discrepancies between central review and institutional diagnoses – most of which were minor, reflecting differing opinions as to tumour grade or subtype.

A larger analysis of frozen paediatric specimens from 17 500 cases banked between 1993 and 2003 showed only 0.4% (70 of 17 500 cases) had ‘problems’ with tumour content. Of these, 43% were deemed to have insufficient tumour, 30% had no tumour and 19% did not have viable tumour. The remaining 8% of cases were for problematic for miscellaneous reasons.

The 10% diagnostic discrepancy rate quoted above does emphasise the need for morphologic review of submitted tissues so that extremely necrotic or contaminated tissue aliquots (‘normal’ with tumour) are not served to investigators. The central review diagnosis is used as the standard in serving investigators.

Confirmation of morphologic viability of tissues is only the first step in assessing the quality of tissues served to investigators. With an increasing emphasis on molecular uses of BPC tissues, efforts to assess the molecular integrity of tissue became necessary. The CHTN has looked at the molecular integrity of its gynaecologic specimens by RT–PCR and DNA and ribosomal RNA electrophoresis performed on ovarian tissue samples (Jewell *et al*, 2002). RT–PCR assay for amplification of the mRNA gene product (177 bp product) of the HPRT housekeeping gene revealed adequate amplification in 70% of ovarian cases. RNA electrophoresis (assessed by visual estimate of the 18s and 28s ribosomal RNA bands by ethidium bromide staining) also revealed RNA to be of good quality with minimal degradation in 70% of ovarian tissues. DNA electrophoresis showed the genomic DNA was of good quality in 100% of ovarian tissues tested. A collection of solid paediatric tumours collected between 2001 and 2003 was also analysed by RT–PCR. Only four out of these 95 cases (4.2%) were deemed to have insufficient or degraded RNA.

Given limitations on amounts of available tissue, preservation on-site of mRNA by timely snap-freezing of tissues in liquid nitrogen becomes paramount. BPC personnel annually train cooperative group personnel at their meetings in the timely freezing of such tissues. Review of quality control data from the BPC database allows for verification of improved institutional performance in this task. The BPC does this by eliciting feedback from investigators on the quality of their tissues (RNA/DNA ‘preservation’) annually. These data are matched to the institutional tissue source, to give institutional feedback on their performance in tissue procurement.

Unfortunately, time and expense limit the extent of molecular surveillance. A limited number of extractions are currently performed on-site for select cancer specimens. DNA quality is measured by PCR amplification of an approximately 950 bp fragment whereas RNA integrity is measured by either RT–PCR or an Agilent 2100 bioanalyzer (Palo AHCA). The Agilent 2100 bioanalyzer uses a disposable RNA chip to determine the concentration and purity/integrity of 12 RNA samples at a time (using as little as 50 ng each) with a total throughput of 25 min. The system permits rapid screening of a RNA preparation for sample degradation and ribosomal RNA contamination.

The BPC is developing a core facility for extracting nucleic acids from a larger number of tissues which will not only provide more molecular quality control data, but centralised extraction should also allow more investigators to be served per unit of tissue. Our experience has been that we can obtain approximately 1–10 *μ*g of RNA per milligram of viable tissue, and 0.5–5 *μ*g of DNA per milligram of viable tissue. These yields can remain constant over a decade or more when tissue is stored long-term in vapour-phase liquid nitrogen freezers, and this is our standard of storage at the BPC.

## INFORMATICS

Informatics (Qualman *et al*, 2003) can facilitate patient registration, specimen tracking, tissue cataloguing, quality assurance, and specimen availability. The ability of databases to organise and present desired information can also aid in tracking informed consent and institutional compliance and be used to generate tissue bank inventory reports to match investigator requests with specimen availability.

### Design objectives for a banking inventory system

The components of a system must appear seamless to allow for efficient data entry, queries and report preparation, and must also allow for rapid deployment of new services. Consideration should also be made for the future, as systems will become increasingly diverse; supporting multiple architectures, platforms, and databases. Exchange of information between different databases at different institutions may also be or become a concern.

Ideally, an informatics system ensures that data are:

- Available over a long period of time
- Maintained in a standardised format (see common data elements below)
- Able to be disseminated to others as needed
- Collected from collaborative sources and combined.

### Common data elements

The Cancer Cooperative Group Chairs along with the National Cancer Institute recognised the need for common data elements across data systems. As a result, the Intergroup Specimen Banking Committee was formed in 1999 to:

identify existing standards, policies and procedures, particularly those in common usage, which would address the needs of intergroup banking, rather than invent new ones. The goal was to deliver a set of recommendations that would require the least amount of work, change and expense to implement, while providing sufficient guidance to the research community to facilitate useful banking efforts in support of correlative science, to avoid unnecessary conflict and to ensure a sufficient standard of quality. (Report of the Intergroup Specimen Banking Committee, 1999)

Rather than establish standards of hardware, operating systems, or applications, the report instead set forth a standard for mandatory common data elements. These common elements provide for such things as uniform reporting, data exchange, and joint analysis.

Entities subject to these guidelines are medical institutions that submit specimens to one or multiple tissue banks/repositories, science review committees, investigators receiving specimens and responsible for submitting results, and statistical centres (Report of the Intergroup Specimen Banking Committee, 1999).

### Minimum data requirements for banking (Report of the Intergroup Specimen Banking Committee, 1999)

- All samples and subsamples must have a unique identifier.
- The system must adhere to standard coding mechanism.
- The system must be able to maintain an up-to-date inventory by unique identifier to include: original and current quantity where specimens have been sent, when (if) they have been returned, and when they have been exhausted. The system must also be able to invalidate samples.
- The database containing information on sample locations (inventory) may reside at the bank and does not need to be accessible by systems outside the bank.
- The system must be able to flag (reserve) specimens for a particular study.
- The system must track the type of informed consent for research that is allowed.
- The system must track specimens and allow for withdrawal of consent.
- The system must be able to report and provide query results across groups to facilitate any combination of a bank per trial, a bank per disease site, or a bank per group per disease site. Projects may require samples accrued on more than one trial.
- The system must provide quality control/checks on imported data.

### Centralised inventory system (Qualman *et al*, 2003)

**Figure 1:**
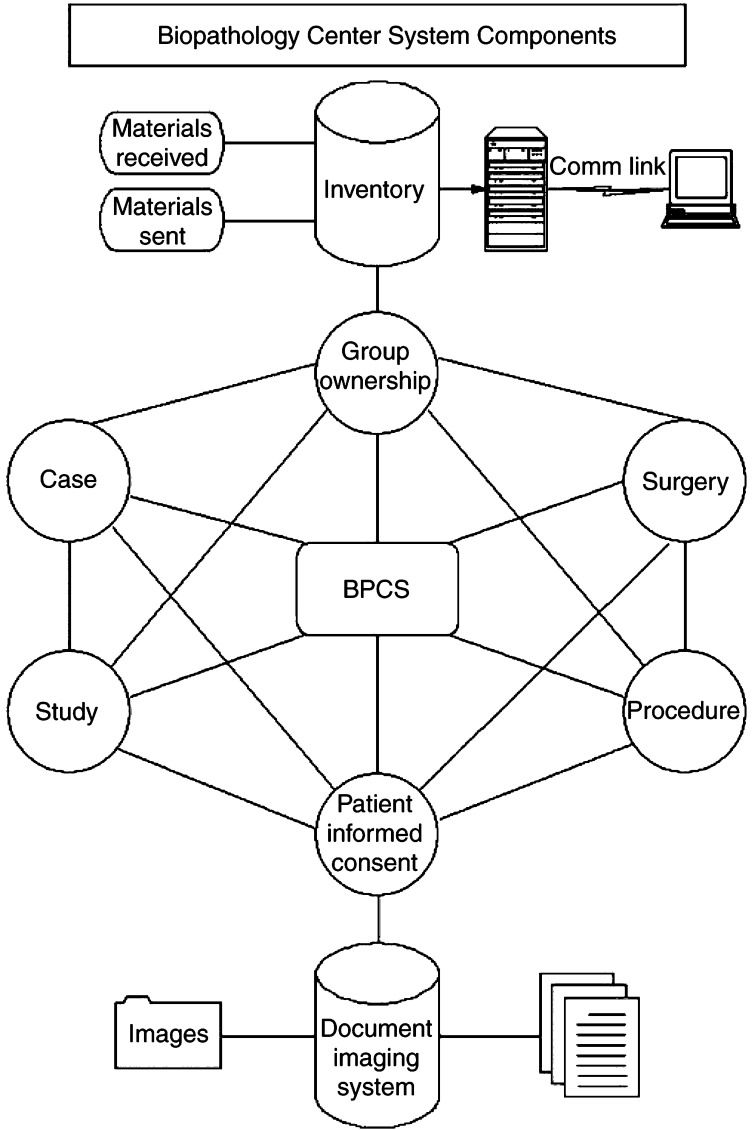
Informatics design of the Biopathology Center System (BPCS). A centralised inventory system is at the heart of the design, which can account for both cooperative group and patient-specific data, while maintaining a real-time inventory of specimen deposits, withdrawals, and residual tissues. Digital images of clinical documents accompanying specimens are created and stored using a document imaging system.

Inventory is at the heart of tissue bank informatics design (Figure 1

). An inventory module is linked to cataloguing, which provides for tagging and tracking of such things as informed consent, group ownership, and tissues reserved or specifically collected for a protocol or study. This ensures that specimens are tracked and distributed appropriately and in compliance with the patient's informed consent, as well as preventing the total depletion of a tissue sample.

The system design also streamlines tissue intake and serving of investigators. Materials are received and processed by a research assistant and entered directly into the system, while specimen shipments are processed through the same interface using a shipment module. The modules keep a precise record of materials as they are received and shipped to produce a real-time inventory of what is available. When specimens are received and inventoried into the central system the specimen is linked back to the specific cooperative group's case file. A full history of the specimen from when it was received to when it was served is maintained per cooperative group per case. Such history gives quick and easy access for reporting purposes.

### Virtual private networks

The use of virtual private networking (VPN) has been fundamental in streamlining the patient enrolment and specimen registration process, reducing repetitive keying of data and ensuring that information is synchronised between physically separate sites. This linking of databases can also be used to create ‘virtual tissue banks’ from the inventory of different sites, thus allowing for optimal matching of requests and distribution of tissues to reach a larger market.

The sensitive nature of medical information makes unauthorised disclosures and data alterations a concern. VPNs can be a cost-effective solution to provide secure point-to-point transactions utilising encryption technologies. The primary requirements for implementation of a secure VPN are:

- Authentication – each end point checks the other and verifies that the transaction belongs to the secure point-to-point site before accepting the transaction or request.
- Strong data encryption to protect sensitive information.
- Transaction privacy (public/private key encryption).
- Encryption using a key unique to each information exchange session.
- Scalability – a VPN needs to be able to grow to accommodate an increase in connected sites.
- Ability to provide audit information.
- Immediate intrusion/attack detection and response – requires continuous evaluation of security policies and practices.

## ETHICS

### Informed consent and confidentiality

Tracking and cataloguing informed consent is the key to banking informatics. Informed consent is the factor by which all tissues are qualified or disqualified for use by a potential researcher. The Intergroup Specimen Banking Committee recently endorsed the use of a three-item checkbox format for summarising levels of informed consent (Report of the Intergroup Specimen Banking Committee, 1999). These levels allow for the patient to designate whether their tissue or case data may be used for: (i) cancer research; (ii) general medical research; and (iii) future patient contact for needed clinical follow-up.

### Linkage and coding

Risks posed to subjects from research with their tissues are strongly related to the identifiability of individual sources of those tissues (Merz *et al*, 1997). Data records which can be directly or indirectly associated with a person's name or other identifying information are referred to as *linked data*. Such identifying elements are date of birth, treating institution, treating physician, medical record numbers, social security numbers, etc. The BPCS uses specialised codes that, by intent, separate the specimen data from the clinical data to maintain patient anonymity. If there is patient consent for the data to be linked, the data sets at the BPC and at a Cooperative Group Statistical Data Center are connected via a virtual private network (see section entitled *Virtual Private Networks*) and decoding can occur.

To permanently de-link data, any codes that will link or identify data or tissues with the donor must be removed, such that even the database manager can no longer trace a tissue or its related data back to the donor.

### Investigator feedback

One metric used to assess the effectiveness of a banking operation is defining ‘who is served and how well’. These questions are answered using an investigator feedback questionnaire process which is performed annually. Despite its apparent success, the CHTN has found through its investigator surveys that not all investigators have received the number of specimens that they desired. In a composite survey of 554 investigators (43% response rate) carried out in 2000 by the CHTN, the following queries with responses were obtained (Table 1

**Table 1:** Responses to a written survey from investigators who have used CHTN tissues

	**Responses (%)**	
**Query**	**Excellent or good**	**Okay or less**
Responsiveness and helpfulness of CHTN staff	94	6
Completeness and accuracy of standard data provided with the specimens (site, tissue type[s], diagnosis)	94	6
How well did the size or weight of specimens received correlate with request as approved by CHTN?	89	11
Was tissue prepared according to specifications as agreed upon with the CHTN?	92	8
Overall quality of tissue received	90	10
Satisfaction with number of specimens received, based upon feedback given by CHTN	74	26
Overall satisfaction with CHTN services	89	11
Importance of the availability of the CHTN tissue to your research	93	7

).

A substantial minority of CHTN investigators, slightly more than one-quarter of them, did not receive the number of specimens they expected. Some of these investigators have unrealistic expectations as to what specimens are available. The dilemma of the CHTN is how to draw the line between their obligation to facilitate research and the risk of frustrating investigators by overestimating which types of specimens can be obtained for their research. ‘Difficult-to-serve’ projects generally have inherent limitations which fall into the following categories:

1. Limitation on amount of tissue available.
2. Limitation based on tissue rarity.
3. Limitation on timing of tissue procurement.
4. Limitation based on tissue consent.

#### Limitation based on amount of tissue available

This is probably the single biggest challenge the CHTN faces in meeting investigator needs. Investigators often ask for multiple aliquots (0.5–1 g) of any given tumour type when those amounts are not realistically available clinically. Advances in screening techniques have led to detection of tumours (e.g. breast cancer) in earlier stages when tumours are generally smaller. Shifts in therapy are leading to fewer surgical resections of certain tumours (e.g. radiation therapy of prostate cancer and chemotherapy/radiation therapy for small cell carcinoma of lung). Bulky end-stage tumours are often not resected, and rapid autopsy (less than 1 h) is rare, such that post-mortem tissues may be less than adequate in quality. Some specimens (e.g. cervical cone biopsies, primary melanoma excisions) may be totally submitted to document minute foci of tumour or depth of invasion. Tissue requests should be for the smallest possible sample size, since the CHTN supplies those before considering large size requests. The largest pool of breast or prostate surgical specimens, for example, are available in aliquots of 0.1–0.25 g or less.

#### Limitations from tissue rarity

The CHTN is not intended as a human tissue bank *per se*, but rather a source of targeted prospective human tissue collection based on investigator need. Limited banking is used to meet specific requests, and rare specimens are banked for longer periods. These are precisely the reasons why the BPC developed banking mechanisms for its cooperative group affiliations.

#### Limitations based on timing of tissue procurement

The issue of time post-surgery can also affect the number of specimens served to an investigator. While the CHTN regularly serves researchers who require rapidly frozen tissue for mRNA preparation, investigator requests for shorter time intervals (e.g. less than 20 min) significantly limit the number of potentially available specimens. Patient diagnosis is the first concern of the CHTN and diagnostic specimens are processed before research specimens. Most surgical specimens are available within 2 h of excision and many within 1 h post-excision. Requesting time intervals under 30 min will seriously limit the number of potential specimens. Also, there is increasing evidence that at least ribosomal RNA is not rapidly degraded following removal of surgical tissues from the human body (Jewell *et al*, 2002). Similarly, it is unusual to see post-mortem intervals of less than 8–12 h; a 3–4 h post-mortem interval is occasionally achieved, but post-mortem intervals which are more rapid can only be achieved by focused specific autopsy protocols facilitated by the clinician.

#### Limitations of tissue availability based on consent

Only tissues that would otherwise be discarded are taken from surgical specimens. Tissues sampled at autopsy must also be within the scope of the signed consent form. Consequently, most ‘normal’ tissues are available from surgical resections to remove diseased and normal adjacent tissue or due to trauma (which are rare). Specific regions of the body will seldom have large areas of ‘normal tissue’ resected surgically (e.g. specific areas of the brain); accordingly, greater quantities of such tissues are more likely to be obtained from autopsy. Ethical problems arise when there is appreciable risk of biopsy for research purposes alone, and costs of research-specific surgical or medical procedures are usually prohibitive. The CHTN is not involved in obtaining tissue removed solely for research purposes; where this is an expectation in a cooperative group study, specific informed consent must be obtained.

It is encouraging to note that overall investigator satisfaction with CHTN services is in the range of 90%. This is because the CHTN is not just a supplier of tissues, but is also an educator on how investigators can increase their project's success, which may include referring the investigator to other tissue resources. When an investigator makes an unrealistic or unreasonable request, a letter is sent to the investigator explaining the problem with their request. The CHTN provides information on alternative technologies or methods (e.g. surgery *vs* autopsy) to serve the request. For rare requests, the CHTN institution serving as primary contact for the investigator informs the investigator in the acceptance letter that the specimens are rare, and estimates how long it might take to fill the request. These requests are also referred immediately to the National Cancer Institute's tissue expediter (301-496-7147 or tissexp@mail.nih.gov) so as to assist the investigator in finding new/other tissue resources.

## FUTURE CHALLENGES

The ultimate role of the pathologist as a tumour banker in this setting is that of both a data miner and tissue refiner (Becich, 2000). One of the chief complaints of investigators concerning tumour banks is the lack of sizeable specimens received for their research. It is incumbent upon the pathologists/bankers, as they review a research proposal, to educate the investigator both on the inherent limitations of tissue acquisition and alternate research techniques that utilise less tissue. Fortunately, with the advent and increased usage of nucleic acid amplification techniques and fluorescent *in situ* hybridisation, such alternate techniques are more readily available; moreover, coincidental use of microarray technologies with nucleic acid amplifications may allow for screening of thousands of genes (Farkas *et al*, 1996). Ultimately, more can be done with less, with careful planning and communication.
